# Population structure and ecotype‐associated genomic differentiation in Bangladeshi rice germplasm (*Oryza sativa* L.)

**DOI:** 10.1002/tpg2.70336

**Published:** 2026-11-11

**Authors:** Asami Tomita, Yuto Makita, Yoshihiko Hirai, M. M. Emam Ahmed, Mohammad Asik Iqbal Khan, Mohammad Abdul Latif, Mohammad Khalequzzaman, Endang M. Septiningsih, Mitsuhiro Obara, Hiroki Saito

**Affiliations:** ^1^ Graduate School of Environmental, Life, Natural Science and Technology Okayama University Okayama Japan; ^2^ Bangladesh Rice Research Institute (BRRI) Gazipur Bangladesh; ^3^ Department of Soil and Crop Sciences Texas A&M University College Station Texas USA; ^4^ Biological Resources and Post‐harvest Division Japan International Research Center for Agricultural Sciences (JIRCAS) Tsukuba Japan; ^5^ Tropical Agriculture Research Front (TARF) JIRCAS Ishigaki Japan

## Abstract

In Bangladeshi rice (*Oryza sativa* L.) germplasm, cultivars or landraces are traditionally classified into four principal ecotypes based on seasonal moisture regimes: Boro, Aman, Aus, and Jhum. The relationships among ecotype differentiation, genomic ancestry, and population structure remain poorly understood. We investigated the genetic diversity and population structure of 333 Bangladeshi rice accessions with respect to their ecotype classification. We identified a total of 25,994 single nucleotide polymorphism (SNP) markers across the whole genome of this panel. From the SNP data and discriminant analysis of principal components, STRUCTURE analysis, genetic diversity analysis, and neighbor‐joining analyses, we identified three groups, corresponding to Aromatic, Indica, and Aus. They differed in both genetic ancestry and degree of ecotype‐associated differentiation. Aus consisted of all four ecotypes and showed clear ecotype‐associated differentiation, suggesting adaptation to distinct ecological environments. Aromatic accessions shared a common genetic background and maintained partial ecotype‐associated differentiation between Aman and Boro. Indica showed reduced genetic diversity and weak ecotype differentiation, reflecting intensive crop improvement and ecotype admixture. The genomic population structure of Bangladeshi rice germplasm was therefore shaped by both ecological adaptation and human‐mediated selection across distinct ancestry groups. Our findings provide deeper insights into the genetic and ecological foundations of this germplasm, supporting its future use in genome‐wide association studies and genomic prediction and its exploitation for rice improvement in Bangladesh and beyond.

## Plain Language Summary

Rice (*Oryza sativa* L.) is an important staple crop in Bangladesh, where rice germplasm is traditionally classified into four ecotypes—Boro, Aman, Aus, and Jhum—based on seasonal cultivation. How these ecotypes relate to the standard global genetic diversity and population structure framework remains unclear. To address this issue, we used 333 rice accessions from Bangladesh and analyzed the corresponding genome‐wide single nucleotide polymorphism data. We identified three major groups, corresponding to Aromatic, Indica, and Aus. The three groups differed not only in genetic ancestry but also in ecotype differentiation. Aus showed clear ecotype‐associated differentiation, suggesting adaptation to different environments. Aromatic showed partial ecotype‐associated differentiation between Aman and Boro. Indica had lower genetic diversity and weaker ecotype differentiation, reflecting intensive crop improvement and ecotype admixture. Our findings support future efforts in genetic and breeding programs in Bangladesh and beyond.

**Abbreviations**
BAM: binary alignment map
BRRI: Bangladesh Rice Research Institute
DAPC: discriminant analysis of principal components
dpMIG‐seq: degenerate oligonucleotide primer multiplexed inter‐simple sequence repeat genotyping by sequencing
GP: genomic prediction
GWAS: genome‐wide association studies
RPAN: 3K Rice Pangenome
SNP: single nucleotide polymorphism
TARF: Tropical Agriculture Research Front
VCF: variant call format

## 1 INTRODUCTION

The genetic diversity and population structure of crop germplasm reflect their evolutionary history, domestication, and adaptation to diverse environments. Understanding crop evolution from wild relatives and their geographic spread provides important insights into the origins of agriculture and crop adaptation (Schreiber et al., 2018). Recent advances in genomic resources and analytical tools have enabled comprehensive analyses of genetic diversity and population structure in diverse crop species (Schreiber et al., 2018). Although modern plant breeding has greatly increased crop productivity, maintaining genetic diversity in breeding populations remains important for sustainable agriculture (Fu, 2015). Therefore, information about the genetic diversity and population structure of crop germplasm is essential for both genetic analyses and crop improvement.

International efforts such as the 3000 Rice Genomes Project (3K RGP) have improved our understanding of Asian rice (*Oryza sativa* L.) diversity and evolutionary history, enabling more efficient use of accessions for rice improvement (Sun et al., 2017; W. Wang et al., 2018). Asian cultivated rice has been classified mainly into Indica and Japonica groups, together with the Aus (circum‐Aus) and Aromatic (circum‐Basmati) groups that are distributed predominantly in South Asia (W. Wang et al., 2018). Previous studies have revealed substantial variation in the population structure of rice genetic resources among countries. For example, temperate Japonica predominates in Japan (Fujino & Shirasawa, 2022); both Indica and Japonica occur in northern Vietnam (Luong et al., 2021); Indica predominates in Cambodia (Orn et al., 2020) and Indonesia (Thomson et al., 2007), with tropical Japonica also in Indonesia; and Madagascar harbors Indica, Japonica, and Malagasy‐specific groups (Ahmadi et al., 2021). Although the rice populations in many regions are composed primarily of Indica and Japonica groups, local adaptation and evolutionary history have resulted in region‐specific population structures.

In Bangladeshi rice germplasm, three major genetic groups—Aus, Indica, and Japonica—have been reported (M. Wang et al., 2013). In Bangladesh, rice is traditionally classified into three principal ecotypes based on seasonal moisture regimes: Aus, Aman, Boro (Parsons et al., 1999), and Jhum (Chakma & Ando, 2008). Aus cultivars are photoperiod insensitive and are grown in upland areas during the pre‐monsoon season. Aman is mostly photoperiod sensitive and is cultivated as a lowland rainfed rice. Boro is grown in the dry season with supplemental irrigation. Jhum rice is cultivated under a slash‐and‐burn agricultural system in the hill regions (Chakma & Ando, 2008). Bangladeshi rice germplasm exhibits high genetic diversity, which is strongly influenced by ecological differentiation among rice ecotypes (Khan et al., 2017). However, previous studies using simple sequence repeat (SSR) markers have provided limited resolution, and the corresponding factors that maintain genetic diversity and the effects of ecotype remain unclear. Moreover, although variations in several phenotypic traits (M. M. E. Ahmed et al., 2021; Khan et al., 2017) have already been evaluated and the identification of associated loci is anticipated, these SSR marker data are insufficient for genome‐wide association studies (GWAS) and genomic prediction (GP).

Our objective here was to determine the population structure and ecotype‐associated differentiation of Bangladeshi rice germplasm by using whole‐genome single nucleotide polymorphism (SNP) data and to assess their implications. To this end, we sequenced 333 accessions, most of which were landraces previously described by Khan et al. (2017), and we classified them into genetic groups on the basis of SNP variation. We then analyzed their genetic diversity and population structure, examined how ecotype differentiation was maintained across different genomic ancestry groups, and compared Bangladeshi accessions with Asian rice cultivars from the 3K RGP (Sun et al., 2017; W. Wang et al., 2018) to contextualize their genetic diversity and its implications. Our results highlight the genetic foundations of how ecological adaptation and breeding history have shaped population structure, providing valuable insights into the genomic variation associated with environmental adaptation.

## 2 MATERIALS AND METHODS

### 2.1 Plant materials

We used a total of 333 rice accessions from Bangladesh, comprising 284 landraces and 49 improved cultivars (Khan et al., 2017) (Table S1). In addition, two reference cultivars, the Japonica cultivar Nipponbare and the Indica cultivar Kasalath, and one introgression line, IR65482‐4‐136‐2‐2 (Pi40 source) (Jeung et al., 2007), were included for comparison. The Bangladeshi accessions were classified into four rice ecotypes: Boro (59 accessions; lowland, irrigated in the dry season, November–May), Aman (171; lowland, rainfed, June–December), Aus (71; upland, rainfed, March–August), and Jhum (32; slash‐and‐burn agriculture in the hill regions, May–September). Nipponbare, Kasalath, and Pi40 source were included as reference cultivars and were not assigned to ecotypes. All of the Bangladeshi accessions were conserved at the Rice Gene Bank, Bangladesh Rice Research Institute (BRRI), Gazipur, Bangladesh, and Tropical Agriculture Research Front, Japan International Research Center for Agricultural Sciences, Ishigaki, Japan.

### 2.2 Library construction, sequencing, and bioinformatics pipeline

To investigate the genetic variation of whole genomes among the Bangladeshi accessions, DNA extraction, library construction, and next‐generation sequencing (NGS) were performed by using the degenerate oligonucleotide primer multiplexed inter‐simple sequence repeat genotyping by sequencing (dpMIG‐seq) method (Nishimura et al., 2024). Genomic DNA from each accession was extracted from a bulk sample of young rice leaves from around 10 individuals.

On the basis of each paired‐end raw read obtained from the dpMIG‐seq, fastp (Chen et al., 2018) was used for trimming and filtering. By using SNAP (Scalable Nucleotide Alignment Program; Zaharia et al., 2011), trimmed reads of each accession were mapped to the Nipponbare reference genome Os‐Nipponbare‐Reference‐IRGSP‐1.0 (IRGSP‐1.0). Sequence alignment/map format files were converted to binary alignment map (BAM) and sorted by using samtools (Li et al., 2009). Variant calling was achieved by using the samtools “mpileup” command (Li et al., 2009) with a set parameter “‐d 0” option for analyses, and then a variant call format (VCF) file was created. SNP loci with heterozygosity rates higher than 20% were removed, and missing genotypes were imputed by assigning the most frequent genotype at each SNP locus for downstream analyses.

### 2.3 Data analysis

To classify the Bangladeshi accessions into groups based on SNP data and to compare the population structure and genetic diversity among groups, discriminant analysis of principal components (DAPCs), analysis of genetic diversity, and phylogenetic tree construction were performed by using “ShiNyP,” an interactive R/Shiny platform that integrates all popular SNP analysis modules within a single and intuitive user interface (Huang et al., 2025). The source code and related resources were obtained from its GitHub repository (https://github.com/TeddYenn/ShiNyP). DAPC was performed to identify distinct genetic clusters of rice accessions. This classification was optimized to maximize genetic differentiation between clusters while minimizing within‐group variation on the basis of Bayesian information criterion values. Genetic diversity in each group was calculated by using diversity metrics for each SNP site, including the SNP‐based nucleotide diversity (*π*), expected heterozygosity (*H_e_*), observed heterozygosity (*H_o_*), and inbreeding coefficient (*F_is_*). From the SNP data, a phylogenetic tree was constructed by using the neighbor‐joining method to clarify the evolutionary relationships among accessions. Mantel's tests (Mantel, 1967) were conducted to investigate the relationship between the geographical distance (in kilometers) and the genetic distance (in Euclidean distance) among the accessions by using the R package vegan (*vegan: Community Ecology Package*; https://github.com/vegandevs/vegan). The distance between the latitude and longitude coordinates of the collection sites for each accession, calculated by using the Haversine formula, was used as the geographical distance. Genetic distance among accessions was calculated as the Euclidean distance on the basis of their SNP genotypes. Pearson's correlation coefficient (*r*) was also calculated, with 9999 permutations for each group.

Additionally, the population structure of the Bangladeshi accessions was estimated with STRUCTURE software v. 2.3.4 (Pritchard et al., 2000). One‐tenth of the total number of SNP markers were selected as core SNPs by using “ShiNyP” (Huang et al., 2025) to retain representative genetic diversity. Ten runs were performed for each number of populations (*K*) set from 1 to 10. Each run had a burn‐in of 50,000 cycles and a Markov chain Monte Carlo replication of 100,000, using an admixture model with correlated allele frequencies. The most probable *K*‐value was determined from the mean log likelihood [ln *P*(*D*)] and delta *K* (Δ*K*) (Evanno et al., 2005), which evaluated the rate of change in ln *P*(*D*) between successive *K*‐values. The optimal *K*‐value was evaluated by using STRUCTURE Harvester (Earl & vonHoldt, 2012).

### 2.4 Merging of SNPs called in the complete RPAN dataset and population differentiation

A total of 453 Asian rice accessions were used from the 3K Rice Pangenome (RPAN) project (Sun et al., 2017) (https://cgm.sjtu.edu.cn/3kricedb/index.php) to compare population structures between the Bangladeshi rice germplasm and the Asian rice accessions. These 453 accessions were selected as high‐quality accessions with sequencing depth >20x and mapping depth >15x and broadly represented the population structure of Asian rice accessions (W. Wang et al., 2018). Whole‐genome sequence reads from each Asian rice accession were mapped to IRGSP‐1.0 by using the same procedure as described above. The BAM files obtained for these 453 accessions were combined with those of the Bangladeshi rice accessions, and variant calling was performed by using the same procedure as described above.

To identify SNPs that were callable in the Bangladeshi dataset, we first filtered the SNPs within 336 Bangladeshi accessions by using the original VCF file. Variants were retained if they were biallelic and had a missing rate (*F_MISSING*) less than 90% and a depth (*DP*) greater than 1. Next, the positions of these filtered SNPs were extracted to generate a list of SNP coordinates. We used this list to select only the corresponding sites from the combined VCF file of Bangladeshi accessions and 453 Asian rice accessions. Subsequently, the combined VCF file was further filtered across all samples to retain SNPs with *F_MISSING* below 5%, *DP* greater than 10, and a minor allele frequency above 5% for downstream analyses. Moreover, to reduce linkage disequilibrium, SNPs were pruned by using a window size of 50 SNPs, a step size of five SNPs, and an *r* ^2^ threshold of 0.2.

We used this merged VCF file to impute missing genotypes in the SNPs by using the column‐wise mean. To correct for potential batch or NGS library effects, SNP‐wise linear regression was performed with the project of origin as the categorical independent variable, and residuals from this regression were retained for subsequent analyses. SNPs with zero variance after residualization were excluded. To compare the population differentiation of the Bangladeshi accessions and Asian rice accessions, pairwise fixation index (*F_ST_*) values were calculated between groups by using the method of Weir and Cockerham (1984) and the R SNPRelate package (Zheng et al., 2012). The Bangladeshi accessions were categorized into the groups classified by DAPC, and the Asian rice accessions were allocated to five groups: Japonica, Aromatic, Admix, Indica, and Aus, as classified by Sun et al. (2017).

## 3 RESULTS

### 3.1 Classification of Bangladeshi rice germplasm

Whole‐genome resequencing was performed on 336 Bangladeshi rice accessions. Two reference accessions, Nipponbare (Japonica) and Kasalath (Indica), and Pi40 source were included for comparison. A total of 25,994 SNPs were retained for downstream analysis after filtering.

For DAPC using a subset of these SNPs, the Bayesian information criterion curve declined rapidly from *K *= 1 to *K* = 3 and then plateaued more gradually, suggesting that *K* = 3 was the optimal number of clusters (Figure S1). Clustering analysis via DAPC partitioned the 336 accessions into 56 in Group 1 and 204 in Group 2, with the remaining 76 being classified into Group 3 (Figure 1). Nipponbare and Kasalath were in Group 3, and Pi40 source was in Group 2 (data not shown). Nipponbare was located toward Group 1 side of Group 3 cluster. It should be noted that Nipponbare was the only temperate Japonica in the panel. The first two discriminant functions explained 77.2% and 22.8% of the total discriminant variance (data not shown). The DAPC biplot, with the plot of accession densities on the discriminant function, showed a clear separation of the 336 accessions into the three clusters, with no accessions overlapping between groups.

**FIGURE 1:**
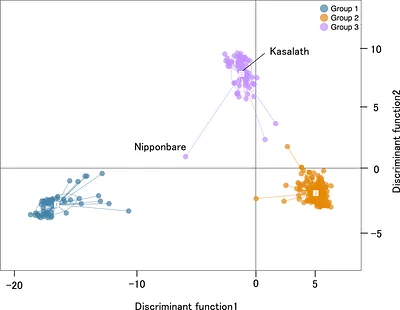
Discriminant analysis of principal components (DAPCs) of Bangladeshi rice germplasm. The accessions of Groups 1, 2, and 3 were indicated by blue, orange, and purple, respectively. Each node represents the genotype of an individual accession, connected to the centroid of the cluster to which the accession was assigned on the basis of *K*‐means clustering of the DAPC scores.

To clarify the correspondence between the classification based on the genotype data from the SNPs and that based on SSR marker data, the accessions in the three groups were compared with those in the three clusters, I, IIa, and IIb, classified by Khan et al. (2017) (Table S2). The accessions classified into Cluster I (mainly the Japonica group) fell mostly into Group 1; those classified into Cluster IIa (Aus group) fell mostly into Group 3, and those classified into Cluster IIb (Indica group) fell mostly into Group 2. These results indicated a high concordance between the SNP‐ and SSR‐based classifications of the accessions and confirmed the robustness of the inferred population structure.

### 3.2 Population structure of Bangladeshi rice germplasm

To evaluate whether the genetic groups identified by DAPC were supported by an independent, model‑based approach, we performed a STRUCTURE analysis of the population structure within the Bangladeshi accessions. For this analysis, 2594 core SNPs among 25,994 imputed SNPs were selected as a representative subset to retain the genetic variation present in the full dataset. The optimal number of clusters (*K*) was determined by using the Δ*K* method to be three (Figure S2). The Bangladeshi rice germplasm comprised three subpopulations, a, b, and c (Figure 2). The subpopulations, a (blue), b (orange), and c (purple), were largely associated with Groups 1, 2, and 3, respectively, although most of the accessions showed genetic admixture among two or three subpopulations. These results indicated that the genetic structure was largely differentiated among the three groups in the Bangladeshi rice germplasm. The observed shared genetic variations among the groups may reflect ancestral polymorphism or hybridization events. Ancestral polymorphisms may have been retained in multiple groups, resulting in shared alleles that can produce an admixture‐like pattern in STRUCTURE without recent hybridization events. The STRUCTURE classification was largely consistent with the clustering pattern obtained from DAPC, while some accessions showed shared genetic ancestry among the three groups. Several accessions positioned away from the main DAPC clusters also showed admixed ancestry in STRUCTURE analysis. The shared genetic variations may reflect ancestral polymorphism or hybridization among the groups.

**FIGURE 2:**
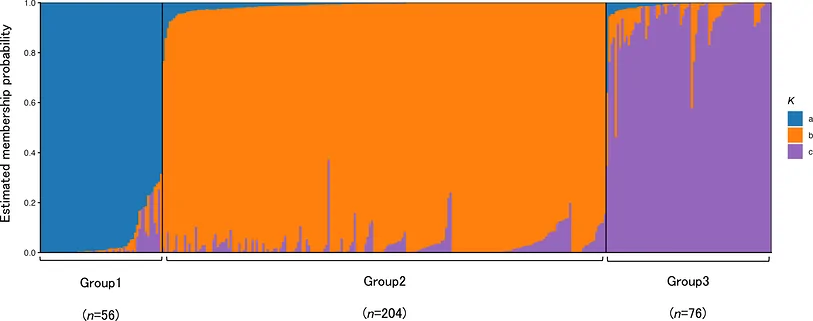
STRUCTURE results of the 336 Bangladeshi rice accessions (mean of 10 replicates) at *K* = 3. Each color represents one subpopulation. Each accession is represented by a vertical bar, and the length of each colored segment in each bar represents the proportion contributed by each subpopulation.

### 3.3 Relationship between population structures of Bangladeshi and Asian rice accessions

To place the three genetic groups identified in the Bangladeshi rice germplasm within a broader Asian rice context, we next compared their genetic relationships with major Asian rice subpopulations. Pairwise *F_ST_* values between the three Bangladeshi groups and the five Asian subgroups classified by Sun et al. (2017) were calculated (Table 1). The values ranged from 0.061 to 0.146. In Group 2, the lowest *F_ST_* values were observed with Indica (0.061) and Admix (0.062), and the highest with Japonica (0.130). In Group 3, the lowest *F_ST_* was observed with Aus (0.063) and the highest was with Japonica (0.146). These results implied that Group 2 corresponded to the Indica group and Group 3 to the Aus group. Group 1 showed the lowest differentiation with Aromatic (0.062) and the highest values with Japonica (0.107), Indica (0.103), and Aus (0.104), suggesting that Group 1 was genetically similar to the Aromatic group rather than to the Japonica group defined among Asian rice accessions.

**TABLE 1:** Pairwise fixation index (*F_ST_*) between three groups classified by DAPCs (discriminant analysis of principal components) and five subpopulations of Asian rice accessions.

Group/subpopulation	Japonica	Aromatic	Admix	Indica	Aus
1	0.107	0.062	0.086	0.103	0.104
2	0.130	0.085	0.062	0.061	0.078
3	0.146	0.090	0.081	0.087	0.063

### 3.4 Genetic diversity in each group of Bangladeshi rice germplasm

To characterize each identified group, we compared the levels of genetic diversity and the composition of landraces and improved cultivars among the three groups by using DAPC (Figure S3). Landraces comprised more than 70% of the accessions in all three groups. The highest frequency of improved cultivars was observed in Group 2 (22%); Group 1 also had a few improved cultivars (7%), whereas no improved cultivars were detected in Group 3.

Genetic diversity analysis revealed that Group 1 had the lowest SNP‐based nucleotide diversity (*π* = 0.2094) and expected heterozygosity (*H_e_* = 0.2057). In contrast, Group 3 had the highest (*π* = 0.2427, *H_e_* = 0.2396) and Group 2 was intermediate (*π* = 0.2244, *H_e_* = 0.2233) (Table 2). Observed heterozygosity (*H_o_*) was highest in Group 3 (0.0788), lowest in Group 2 (0.0565), and intermediate in Group 1 (0.0684). The inbreeding coefficient (*F_is_*) was highest in Group 2 (0.7468), indicating a high level of inbreeding, whereas Groups 1 and 3 had lower *F_is_* values (0.6673 and 0.6708, respectively), suggesting lower levels of inbreeding. Group 1 accessions exhibited low genetic diversity but did not show high inbreeding. In contrast, Group 2 showed intermediate genetic diversity but high inbreeding due to intensive selection or improvement. Group 3 maintained the highest genetic diversity among the three groups, with low levels of inbreeding. These results suggested that the three groups had experienced different evolutionary histories: The patterns may reflect the effects of selection or genetic bottlenecks in Group 1, crop improvement in Group 2, and traditional landrace diversity in Group 3.

**TABLE 2:** Diversity parameters of the identified groups in DAPCs (discriminant analysis of principal components) of Bangladeshi rice accessions.

Group	*n*	*π*	*H_e_*	*H_o_*	*F_is_*
1	56	0.2094	0.2057	0.0684	0.6673
2	204	0.2244	0.2233	0.0565	0.7468
3	76	0.2427	0.2396	0.0788	0.6708

*Note*: *π*, SNP‐based nucleotide diversity*; H_e_*, expected heterozygosity*; H_o_*, observed heterozygosity*; F_is_*, inbreeding coefficient.

### 3.5 Ecotype‐associated genomic structure across genetic groups

To investigate the relationship between ecological classification and the genomic structure of the germplasm, we examined the relationships among ecotypes, genetic groups, and geographic distance. Group compositions in each ecotype were compared to characterize the four ecotypes (Figure 3). More than 50% of the accessions in Boro, Aman, and Aus were assigned to Group 2. In Group 1, Boro (15%) and Aman (25%) were frequent, whereas Aus (5%) and Jhum (3%) were less frequent. On the other hand, a higher proportion of the accessions of Aus (44%) and Jhum (66%) were classified as Group 3, compared with Boro (19%) and Aman (6%). These results indicated that the ecotype distribution was not random across the three groups. Instead, the Boro and Aman accessions were associated predominantly with Group 2; the Jhum accessions were enriched in Group 3, whereas Aus accessions were associated mostly with both Groups 2 and 3, suggesting that there was a nonuniform association between ecological classification and genomic ancestry.

**FIGURE 3:**
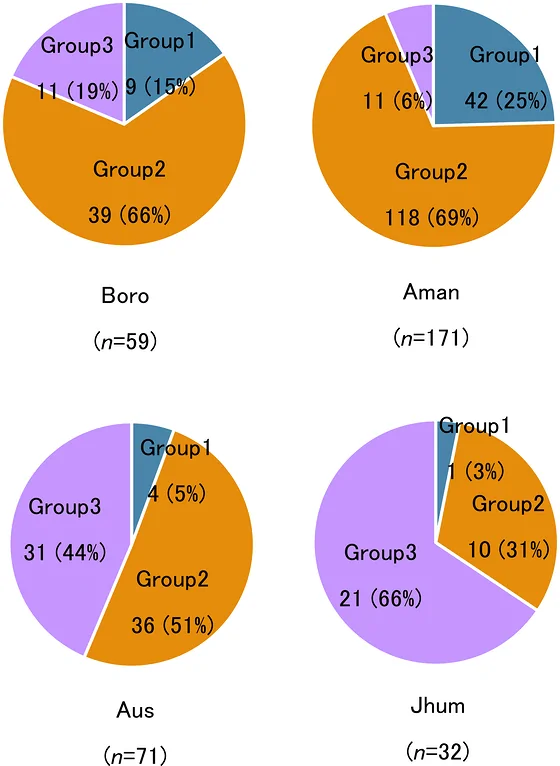
Ecotype differentiation across the three groups classified by DAPCs (discriminant analysis of principal components), categorized into four ecotypes: Boro, Aman, Aus, and Jhum. Boro: lowland, irrigated in the dry season (November–May); Aman: lowland, rainfed (June–December); Aus: upland, rainfed (March–August); and Jhum: slash‐and‐burn agriculture in hill regions (May–September).

To clarify the evolutionary relationships among the Bangladeshi rice accessions, a phylogenetic tree was constructed by using the neighbor‐joining method (Figure 4). The distribution of ecotypes across the phylogeny was then examined to evaluate the relationship between ecological classification and genetic ancestry. Most of the accessions formed three major, distinct clades, which were associated with the three groups and characterized by short genetic distances among them, suggesting that there was a shared genetic background or origin within each group. Nipponbare was located in the same clade as Group 1 accessions, even though it was classified into Group 3 in the DAPC. Ecotypes were broadly distributed across the phylogeny, rather than being restricted to specific major clades. However, differences in ecotype enrichment were observed among the three groups. All four ecotypes were widely distributed across the major clade of Group 2, indicating genetic admixture in Group 2. In Group 3, the ecotypes showed a distinct distribution, with clustering among the four. In Group 2, Aus and Jhum tended to be associated with distinct subclades. In Group 1, Aman had a prominent subclade. These results suggested that ecological classification in the Bangladeshi germplasm was not phylogenetically constrained but was partially superimposed on genetic ancestry.

**FIGURE 4:**
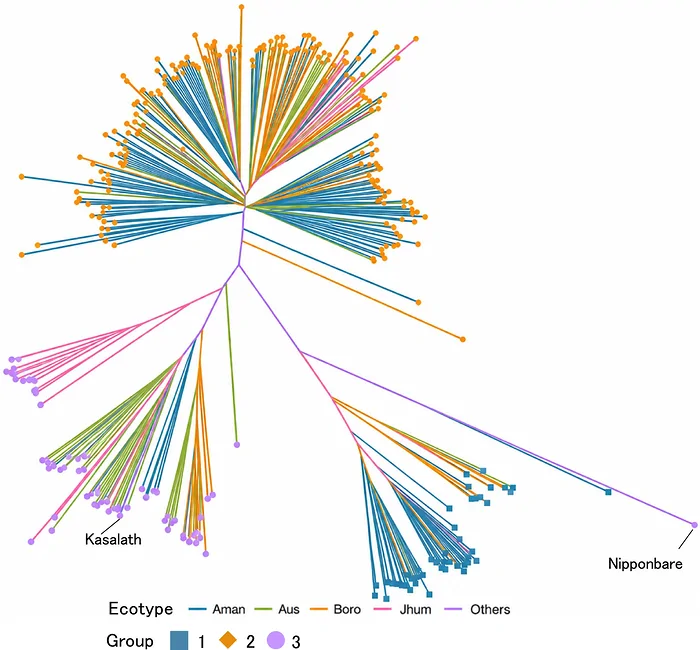
Phylogenetic relationship of Bangladeshi rice germplasm with ecotypes and groups. The line colors indicate assignment to the four ecotypes (Boro, Aman, Aus, and Jhum), and the symbols at the tips of the phylogenetic tree branches indicate the three DAPCs (discriminant analysis of principal components) groups.

To clarify the relationship between geological and genetic distance within each group, Mantel's test was performed by using the SNP‐based genetic distance and the geographic distance of landrace accessions (Figure 5). A positive correlation coefficient (*r*) between them was observed in Group 3 (*r *= 0.385, *p* = 0.0001). By contrast, weak correlation was found in Group 2 (*r *= 0.082, *p = *0.006) and in Group 1 (*r *= 0.222, *p = *0.0005). These results suggested that the relationship between geographic and genetic differentiation differed among genetic groups. Group 3 exhibited stronger spatial genetic structure than Groups 1 and 2, suggesting that isolation by distance might have contributed to the population structure in Group 3. In contrast, Groups 1 and 2 appeared to have been less influenced by geographic isolation, indicating that there had been historical gene flow or human‐mediated movement of the Bangladeshi germplasm.

**FIGURE 5:**
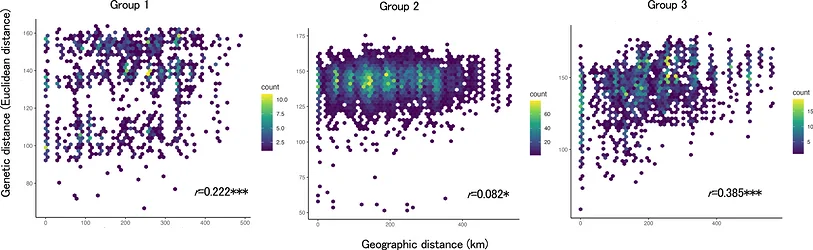
Spatial genetic structure within ecotype‐associated genetic groups. Mantel's tests of the relationship between geographical distance (in kilometers) and genetic distance (Euclidean distance) among the rice landrace accessions were conducted. The distance between the latitude and longitude coordinates of the collection sites for each accession, using the Haversine formula, was used as the geographical distance. Genetic distance among accessions was calculated as the Euclidean distance based on the SNP genotypes of the accessions. Significance levels are indicated as follows: *p < 0.05 and ***p < 0.001 on the basis of permutation tests under the null hypothesis of no correlation.

## 4 DISCUSSION

To understand how ecological rice ecotypes contributed to genomic differentiation in the Bangladeshi rice germplasm, we examined the relationships among ancestry groups, ecotype classifications, and geographic structure by using a panel of 333 rice accessions and 25,994 SNP markers. We used the SNP data to perform a clustering analysis via DAPC, and we classified the rice germplasm into three distinct groups (Figure 1). The results of the STRUCTURE analysis supported this classification (Figure 2). Bangladeshi rice germplasm has previously been classified mainly into Japonica, Indica, and Aus groups (M. Wang et al., 2013). Consistent with the previous classification of SSR markers (Khan et al., 2017), the genetic background of Group 1 is primarily Japonica, that of Group 2 is Indica, and that of Group 3 is Aus. *F_ST_* between the three Bangladeshi groups and the five Asian subgroups (Sun et al., 2017) indicated that Group 2 corresponded to the Indica group and Group 3 to the Aus group (Table 1). However, Group 1 showed greater genetic similarity to the Aromatic group than to the Japonica group defined among the Asian rice accessions. The Aromatic group consists mainly of South Asian accessions (Sun et al., 2017; W. Wang et al., 2018) and is thought to have originated primarily from Japonica, with gene flow from the Aus group (Choi et al., 2020; Civáň et al., 2015). In the Aromatic group, the *Wx* (Z. Y. Wang et al., 1995) locus, which affects amylose content and stickiness on cooking, the *Badh2.1* (Chen et al., 2008) locus for rice fragrance, and their surrounding genomic regions are highly diverse, suggesting that they have complex selection histories related to grain quality (W. Wang et al., 2018). Therefore, the accessions in Group 1 of the Bangladeshi rice germplasm may share an evolutionary background with the Aromatic group, and this may have been involved in historical selection for grain quality traits, including fragrance. In Bangladesh, farmers have traditionally maintained and cultivated aromatic landraces repeatedly because they preferred grain quality, including fragrance and cooking quality (Kretzschmar et al., 2018; Rayhan et al., 2023). The repeated cultivation and maintenance of aromatic landraces may have contributed to the low genetic diversity in Group 1. However, its *F_is_* was relatively low, indicating that low genetic diversity was not necessarily associated with a strong deficiency of heterozygote in Group 1. The aromatic group has experienced gene flow from Aus (Choi et al., 2020; Civáň et al., 2015). The genetic differentiation between Aus and Aromatic varies across the regions. In previous reports from other Asian regions, including northern Vietnam (Luong et al., 2021) and Cambodia (Orn et al., 2020), the Aus and Aromatic groups were not clearly differentiated, whereas they were clearly differentiated in Indonesia (Thomson et al., 2007). We revealed here that the Aus (group 3) and Aromatic (group 1) groups formed distinct clusters (Figures 1 and 4). The regional differentiation between Aus and Aromatic groups in Bangladeshi rice germplasm might reflect local adaptation or breeding history.

To examine the ecological and geographical factors shaping the population structure of Bangladeshi rice germplasm, we compared the ecotype composition, phylogenetic relationships, and spatial genetic patterns among the three groups. The accessions in Group 3 showed clear ecotype‐specific differentiation (Figures 3 and 4). Although all four ecotypes were distributed in Group 3, our phylogenetic analysis revealed the presence of partial ecotype‐associated subclades, and Mantel's test confirmed a significant correlation between geographic and genetic distance in this group (Figure 5). These results suggested that both ecological differentiation and spatial isolation have contributed to the genomic population structure of Group 3. In Bangladeshi rice cultivation, rice is traditionally classified into four kinds of ecotype—Aus, Aman, Boro, and Jhum—on the basis of the seasonal moisture regime (Chakma & Ando, 2008; Parsons et al., 1999). These ecotype‐specific landraces had been adapted to distinct environments. Aman is photoperiod sensitive and has a long growth period, whereas Boro is photoperiod insensitive and is cultivated under winter conditions with sufficient water (Parsons et al., 1999). These seasonal differences may limit gene flow among the ecotypes. Additionally, slash‐and‐burn agriculture, known as Jhum, was the traditional farming system practiced by local ethnic communities in the Chittagong Hill Tracts of southeastern Bangladesh and was ecologically separated from lowland rice cultivation (Ramakrishnan, 1984). In our analysis, Jhum was predominantly assigned to Group 3 (66%) and Group 2 (31%) (Figures 3 and 4). The Chittagong Hill Tracts are geographically close to mountainous northern and western Myanmar, where Indica‐group landraces have also been reported (Wunna et al., 2016). Thus, the predominance of Aus and Indica group backgrounds in Jhum may reflect the genetic diversity of the Chittagong Hill Tracts. Aus is adapted to a wide range of environments from upland to lowland, and it might have contributed to the differentiation of Boro for adaptation to lowland (Glaszmann, 1987; Lyu et al., 2014). These findings suggest that ecological adaptation, cultivation systems, and geographic isolation may have contributed to the maintenance of the ecotype‐associated genomic differences in Group 3.

Interestingly, an ecotype‐associated subclade was also partially observed in the Aromatic group (group 1) (Figure 4). This finding suggests that ecotype differentiation was not restricted to the Aus group but also occurred in the Aromatic group. Despite the shared ancestry of these groups, ecological adaptation or seasonal isolation may have contributed to partial ecotype‐associated genomic differentiation within the Aromatic group. Interestingly, Mantel's test showed that the relationship between geographic and genetic distances in Group 1 was weaker than that observed in Group 3 (Figure 5). The scatter plot for Group 1 showed two distinct patterns: some geographically distant pairs had small genetic distances, whereas some geographically close pairs had relatively large genetic distances. For example, several of the geographically distant but genetically similar pairs were Aman accessions, while genetically divergent pairs were also observed among accessions of Aman. These patterns suggest that geographic distance does not fully explain the genetic relationships or ecotype in Group 1. The geographically distant but genetically similar accessions may reflect the maintenance or distribution of aromatic germplasm across different regions, whereas the occurrence of genetically divergent accessions in geographically close areas may indicate the coexistence of distinct lineages within the same region.

To infer the evolutionary histories that have shaped the genomic structure of Bangladeshi germplasm, we examined the differences in genetic diversity, ecotype composition, and the history of selection and crop improvement. Group 2 had an admixture of the four ecotypes in the phylogenetic tree (Figure 4), indicating that it had weaker ecotype differentiation than Group 3. In addition, the highest frequency of improved cultivars was observed in Group 2, which corresponded to the Indica group (Figure S3). During the Green Revolution, the International Rice Research Institute developed the Indica‐group semi‐dwarf cultivar IR20, which was the first high‐yielding cultivar released in Bangladesh. Until now, BRRI has developed modern inbred rice cultivars with an Indica‐group genetic background for cultivation in Bangladesh (M. E. Ahmed et al., 2022). Therefore, the genetic structure of Group 2 may reflect historical admixture among ecotypes and intensive human‐mediated breeding in the Indica group, and it may have weakened the ecotype‐associated genomic differentiation within the Indica group (group 2).

To explain apparent differences among the results of the different analyses, we considered each analytical method and SNP dataset used. DAPC analysis was performed using the full set of 25,994 imputed SNPs, whereas STRUCTURE analysis was performed using 2594 core SNPs selected from the same imputed dataset. Despite the difference in the number of SNPs, the major population structure was broadly consistent between DAPC and STRUCTURE, with three major groups identified in both analyses (Figures 1 and 2). The STRUCTURE analysis showed that most accessions had genetically admixed backgrounds across two or three subpopulations (Figure 2). Although Nipponbare was assigned to the Group 1 side of Group 3 cluster in DAPC, our phylogenetic analysis placed it within the Japonica group clade together with Group 1 accessions (Figure 4). These results reflect the fact that DAPC captures multivariate differentiation rather than phylogenetic relationships. DAPC emphasizes allele‐frequency differentiation among predefined clusters and may not necessarily reflect phylogenetic ancestry. Therefore, several accessions might share allele‐frequency patterns with those of another group, despite different evolutionary origins.

Although previous studies have constructed pangenomes for the Aus group in Asian rice (Sun et al., 2017; W. Wang et al., 2018), our ecotype clustering of Group 3 suggests that Bangladeshi rice germplasm may harbor region‐specific genomic variations associated with ecological adaptation. They might include ecotype‐specific accessory genes for adaptation, as well as both fixed and introgressed regions, such as structural variation, copy number variation, and presence–absence variation. However, a single reference genome has limitations in detecting these mutations. To clarify the actual genome sequence of Bangladeshi rice germplasm, future pangenome analyses of these accessions may help us to identify the accessory genomic variation associated with ecotype differentiation and environmental adaptation.

The population structure identified here has important implications for downstream genetic analyses and breeding, including GWAS, GP, and hybridization strategies. Bangladeshi rice germplasm was structured into three groups—Aromatic (group 1), Indica (group 2), and Aus (group 3)—with distinct evolutionary histories and degrees of ecotype‐associated differentiation. This population structure may strongly influence GWAS and GP owing to the different evolutionary histories and selective pressures. In rice, mixing subpopulations with different genetic backgrounds could improve the detection of causal variants in GWAS by increasing the sample size; however, it may also yield large numbers of false positives associated with population structure (Hamazaki et al., 2020). Accounting for the confounding effects of population structure in an analysis requires selection of the model (Zhao et al., 2011), analysis of each subpopulation (Lee et al., 2024; Xu et al., 2016), or GWAS meta‐analysis (Yang et al., 2025). In particular, the ecotype‐associated differentiation in Group 3 and the extensive admixture observed in Group 2 indicated that both ancestry and ecology‐related structure should be considered in association analyses of Bangladeshi rice accessions. Several phenotypic traits, including rice blast resistance (Khan et al., 2017) and root development traits under different nitrogen concentrations, and their forms (M. M. E. Ahmed et al., 2021), have already been evaluated in this germplasm. Combining the SNP data from our study with these phenotype data from GWAS or GP analyses will make it possible to select promising accessions and elucidate the associated genetic loci.

Previous studies have revealed different aspects of the population structure of Bangladeshi rice germplasm. M. Wang et al. (2013) identified differentiation among groups such as Indica, Aus, and Aromatic. In another study, Khan et al. (2017) characterized the germplasm primarily into two major groups, such as Indica and Japonica. The present study analyzes the same set of germplasm as Khan et al. (2017). However, they used only 74 SSR markers and mainly analyzed population structure. In contrast, the high‐density SNP dataset in the present study provided a higher‐resolution view of the genomic structure of this germplasm panel. Additionally, our analysis based on 25,994 SNP markers characterized the relationships between genetic groups and ecotypes in detail. We revealed clear differentiation between the Aromatic and Aus groups and identified distinct patterns of genetic diversity, ecotype composition, and relationships of geographic and genetic distance among the three groups. These findings provide new insights into the population structure and ecotype differentiation of Bangladeshi rice germplasm.

## 5 CONCLUSION

We used 25,994 SNP markers to analyze the genetic diversity and population structure of a panel of 333 rice accessions from Bangladesh. From the whole‐genome SNP data, these accessions were classified into three genomic groups: Aromatic, Indica, and Aus. The three groups differed not only in their genetic ancestry but also in their degree of ecotype‐associated genomic differentiation. The Aus group (group 3) comprised mainly four rice ecotypes (Boro, Aman, Aus, and Jhum) and showed clear ecotype‐associated differentiation, suggesting adaptation to different environments. Aromatic group (group 1) accessions shared a common genetic background, and partial ecotype‐associated differentiation between Aman and Boro was maintained within the group. By contrast, the Indica group (group 2) exhibited weaker ecotype differentiation, reflecting intensive selection during crop improvement and ecotype admixture. Thus, the genomic structure of Bangladeshi rice germplasm was formed by both ecological adaptation and breeding history across distinct genetic groups. By highlighting the relationships among genetic ancestry, ecotype‐associated differentiation, and diversity within the germplasm, our study provides in‐depth insights into the genetic foundations of the future use of this germplasm in GWAS, GP, and rice breeding programs in Bangladesh and beyond.

## AUTHOR CONTRIBUTIONS

**Asami Tomita**: Conceptualization; data curation; formal analysis; funding acquisition; investigation; methodology; project administration; supervision; validation; visualization; writing—original draft; writing—review and editing. **Yuto Makita**: Methodology; validation; writing—review and editing. **Yoshihiko Hirai**: Validation; writing—review and editing. **M. M. Emam Ahmed**: Validation; writing—review and editing. **Mohammad Asik Iqbal Khan**: Resources; writing—review and editing. **Mohammad Abdul Latif**: Resources; writing—review and editing. **Mohammad Khalequzzaman**: Resources; writing—review and editing. **Endang M. Septiningsih**: Validation; writing—review and editing. **Mitsuhiro Obara**: Validation; writing—review and editing. **Hiroki Saito**: Resources; validation; writing—review and editing.

## CONFLICT OF INTEREST STATEMENT

The authors declare no conflicts of interest.

## Supporting information




**Figure S1**. Graph of Baysian information criterion vs. number of clusters, indicating the optimum number of clusters (K = 1 to 30) inferred in the Bangladeshi accessions for DAPC.

**Figure S2**. Plot of ΔK and mean ln P(D) against K = 1 to 10, with 10 runs performed for each K in the STRUCTURE analysis.

**Figure S3**. Composition of rice accessions among three groups calssified on the basis of the DAPC analysis of SNP data, when categorized into imorived varieties, landraces, and others. Improved: variety bred after the Green Revolution.

**Table S1**. Accessions used in this study.

**Table S2**. Composition of rice accessions in three groups classified on the basis of DAPC analysis of SNP data, when categorized into three clusters (Im IIa, and IIb) on the basis of SSR marker data (Khan et al., 2017), ant others.

## Data Availability

The fastq file used in this study is available from the NCBI Sequence Read Archive (SRA) under BioProject accession number PRJNA1469431.

## References

[tpg270336-bib-0001] Ahmadi, N. , Ramanantsoanirina, A. , Santos, J. D. , Frouin, J. , & Radanielina, T. (2021). Evolutionary processes involved in the emergence and expansion of an atypical *O. sativa* group in Madagascar. Rice, 14, Article 44. 10.1186/s12284-021-00479-8 PMC8137759 34014423

[tpg270336-bib-0002] Ahmed, M. E. , Biswas, A. , & Afrin, S. (2022). Contribution of IR20 and IR64 in developing three Bangladeshi popular rice cultivars. Plant Breeding and Biotechnology, 10, 81–93. 10.9787/PBB.2022.10.2.81

[tpg270336-bib-0003] Ahmed, M. M. E. , Iqbal Khan, M. A. , Kabir, S. , Fukuta, Y. , & Obara, M. (2021). Genetic variations of root development traits under different concentrations and forms of nitrogen in Bangladeshi rice (*Oryza sativa* L.) accessions. Soil Science and Plant Nutrition, 67, 31–40. 10.1080/00380768.2020.1843370

[tpg270336-bib-0004] Chakma, S. S. , & Ando, K. (2008). Jhum cultivation in Khagrachari hill district of Bangladesh—A subsistence farming practices in ethnic minorities. Journal of Agroforestry and Environment, 2, 1–8.

[tpg270336-bib-0005] Chen, S. , Yang, Y. , Shi, W. , Ji, Q. , He, F. , Zhang, Z. , Cheng, Z. , Liu, X. , & Xu, M. (2008). *Badh2*, encoding betaine aldehyde dehydrogenase, inhibits the biosynthesis of 2‐acetyl‐1‐pyrroline, a major component in rice fragrance. The Plant Cell, 20, 1850–1861. 10.1105/tpc.108.058917 PMC2518245 18599581

[tpg270336-bib-0006] Chen, S. , Zhou, Y. , Chen, Y. , & Gu, J. (2018). fastp: An ultra‐fast all‐in‐one FASTQ preprocessor. Bioinformatics, 34, i884–i890. 10.1093/bioinformatics/bty560 PMC6129281 30423086

[tpg270336-bib-0007] Choi, J. Y. , Lye, Z. N. , Groen, S. C. , Dai, X. , Rughani, P. , Zaaijer, S. , Harrington, E. D. , Juul, S. , & Purugganan, M. D. (2020). Nanopore sequencing‐based genome assembly and evolutionary genomics of circum‐basmati rice. Genome Biology, 21, Article 21. 10.1186/s13059-020-1938-2 PMC7001208 32019604

[tpg270336-bib-0008] Civáň, P. , Craig, H. , Cox, C. J. , & Brown, T. A. (2015). Three geographically separate domestications of Asian rice. Nature Plants, 1, Article 15164. 10.1038/nplants.2015.164 PMC4900444 27251535

[tpg270336-bib-0009] Earl, D. A. , & vonHoldt, B. M. (2012). STRUCTURE HARVESTER: A website and program for visualizing STRUCTURE output and implementing the Evanno method. Conservation Genetics Resources, 4, 359–361. 10.1007/s12686-011-9548-7

[tpg270336-bib-0010] Evanno, G. , Regnaut, S. , & Goudet, J. (2005). Detecting the number of clusters of individuals using the software STRUCTURE: A simulation study. Molecular Ecology, 14, 2611–2620. 10.1111/j.1365-294X.2005.02553.x 15969739

[tpg270336-bib-0011] Fu, Y. B. (2015). Understanding crop genetic diversity under modern plant breeding. Theoretical and Applied Genetics, 128, 2131–2142. 10.1007/s00122-015-2585-y PMC4624815 26246331

[tpg270336-bib-0012] Fujino, K. , & Shirasawa, K. (2022). Fine‐scale genetic structure of the rice landrace population in Japan. Molecular Genetics and Genomics, 297, 711–718. 10.1007/s00438-022-01880-8 35290520

[tpg270336-bib-0013] Glaszmann, J. C. (1987). Isozymes and classification of Asian rice varieties. Theoretical and Applied Genetics, 74, 21–30. 10.1007/BF00290078 24241451

[tpg270336-bib-0014] Hamazaki, K. , Kajiya‐Kanegae, H. , Yamasaki, M. , Ebana, K. , Yabe, S. , Nakagawa, H. , & Iwata, H. (2020). Choosing the optimal population for a genome‐wide association study: A simulation of whole‐genome sequences from rice. The Plant Genome, 13, e20005. 10.1002/tpg2.20005 PMC12807435 33016626

[tpg270336-bib-0015] Huang, Y.‐H. , Chen, L.‐Y. , Septiningsih, E. M. , Kao, P.‐H. , & Kao, C.‐F. (2025). ShiNyP: Unlocking SNP‐based population genetics—An AI‐assisted platform for rapid and interactive visual exploration. Molecular Biology and Evolution, 42(6), msaf117. 10.1093/MOLBEV/MSAF117 PMC12202752 40418736

[tpg270336-bib-0016] Jeung, J. U. , Kim, B. R. , Cho, Y. C. , Han, S. S. , Moon, H. P. , Lee, Y. T. , & Jena, K. K. (2007). A novel gene, *Pi40(t)*, linked to the DNA markers derived from NBS‐LRR motifs confers broad spectrum of blast resistance in rice. Theoretical and Applied Genetics, 115, 1163–1177. 10.1007/s00122-007-0642-x 17909744

[tpg270336-bib-0017] Khan, M. A. I. , Latif, M. A. , Khalequzzaman, M. , Tomita, A. , Ali, M. A. , & Fukuta, Y. (2017). Genetic variation in resistance to blast (*Pyricularia oryzae* Cavara) in rice (*Oryza sativa* L.) germplasms of Bangladesh. Breeding Science, 67, 493–499. 10.1270/jsbbs.17039 PMC5790043 29398943

[tpg270336-bib-0018] Kretzschmar, T. , Mbanjo, E. G. N. , Magalit, G. A. , Dwiyanti, M. S. , Habib, M. A. , Diaz, M. G. , Hernandez, J. , Huelgas, Z. , Malabayabas, M. L. , Das, S. K. , & Yamano, T. (2018). DNA fingerprinting at farm level maps rice biodiversity across Bangladesh and reveals regional varietal preferences. Scientific Reports, 8, Article 14920. 10.1038/s41598-018-33080-z PMC6175857 30297917

[tpg270336-bib-0019] Lee, S. Y. , Lee, G. , Han, J. , Ha, S.‐K. , Lee, C.‐M. , Kang, K. , Jin, M. , Suh, J.‐P. , Jeung, J.‐U. , Mo, Y. , & Lee, H.‐S. (2024). GWAS analysis reveals the genetic basis of blast resistance associated with heading date in rice. Frontiers in Plant Science, 15, 1412614. 10.3389/fpls.2024.1412614 PMC11148375 38835858

[tpg270336-bib-0020] Li, H. , Handsaker, B. , Wysoker, A. , Fennell, T. , Ruan, J. , Homer, N. , Marth, G. , Abecasis, G. , Durbin, R. , & 1000 Genome Project Data Processing Subgroup . (2009). The sequence alignment/map format and SAMtools. Bioinformatics, 25, 2078–2079. 10.1093/bioinformatics/btp352 PMC2723002 19505943

[tpg270336-bib-0021] Luong, N. H. , Linh, L.‐H. , Shim, K.‐C. , Adeva, C. , Lee, H.‐S. , & Ahn, S.‐N. (2021). Genetic structure and geographical differentiation of traditional rice (*Oryza sativa* L.) from Northern Vietnam. Plants, 10, 2094. 10.3390/plants10102094 PMC8540186 34685903

[tpg270336-bib-0022] Lyu, J. , Li, B. , He, W. , Zhang, S. , Gou, Z. , Zhang, J. , Meng, L. , Li, X. , Tao, D. , Huang, W. , Hu, F. , & Wang, W. (2014). A genomic perspective on the important genetic mechanisms of upland adaptation of rice. BMC Plant Biology, 14, Article 160. 10.1186/1471-2229-14-160 PMC4074872 24920279

[tpg270336-bib-0023] Mantel, N. (1967). The detection of disease clustering and a generalized regression approach. Cancer Research, 27, 209–220. 6018555

[tpg270336-bib-0024] Nishimura, K. , Kokaji, H. , Motoki, K. , Yamazaki, A. , Nagasaka, K. , Mori, T. , Takisawa, R. , Yasui, Y. , Kawai, T. , Ushijima, K. , Yamasaki, M. , Saito, H. , Nakano, R. , & Nakazaki, T. (2024). Degenerate oligonucleotide primer MIG‐seq: An effective PCR‐based method for high‐throughput genotyping. The Plant Journal, 118, 2296–2317. 10.1111/tpj.16708 38459738

[tpg270336-bib-0025] Orn, C. , Saito, H. , Khan, M. A. I. , Nhuiyan, M. R. , Vathany, T. , Khay, S. , Makara, O. , & Fukuta, Y. (2020). Genetic variation of rice (*Oryza sativa* L.) germplasm in Cambodia. Breeding Science, 70, 576–585. 10.1270/JSBBS.20052 PMC7878939 33603554

[tpg270336-bib-0026] Parsons, B. J. , Newbury, H. J. , & Jackson, M. T. (1999). The genetic structure and conservation of aus, aman and boro rices from Bangladesh. Genetic Resources and Crop Evolution, 46, 587–598. 10.1023/A:1008749532171

[tpg270336-bib-0027] Pritchard, J. K. , Stephens, M. , & Donnelly, P. (2000). Inference of population structure using multilocus genotype data. Genetics, 155, 945–959. 10.1093/genetics/155.2.945 PMC1461096 10835412

[tpg270336-bib-0028] Ramakrishnan, P. S. (1984). The science behind rotational bush fallow agriculture system (jhum). Proceedings/Indian Academy of Sciences, 93, 379–400. 10.1007/BF03053090

[tpg270336-bib-0029] Rayhan, M. U. , Shozib, H. B. , Azam, F. M. S. , & Islam, T. (2023). Variability in 2‐acetyl‐1‐pyrroline production and associated mutations in *BADH2* gene in aromatic rice cultivars of Bangladesh. Gene Reports, 33, Article 101847. 10.1016/j.genrep.2023.101847

[tpg270336-bib-0030] Schreiber, M. , Stein, N. , & Mascher, M. (2018). Genomic approaches for studying crop evolution. Genome Biology, 19, Article 140. 10.1186/s13059-018-1528-8 PMC6151037 30241487

[tpg270336-bib-0031] Sun, C. , Hu, Z. , Zheng, T. , Lu, K. , Zhao, Y. , Wang, W. , Shi, J. , Wang, C. , Lu, J. , Zhang, D. , Li, Z. , & Wei, C. (2017). RPAN: Rice pan‐genome browser for ∼3000 rice genomes. Nucleic Acids Research, 45, 597–605. 10.1093/nar/gkw958 PMC5314802 27940610

[tpg270336-bib-0032] Thomson, M. J. , Septiningsih, E. M. , Suwardjo, F. , Santoso, T. J. , Silitonga, T. S. , & McCouch, S. R. (2007). Genetic diversity analysis of traditional and improved Indonesian rice (*Oryza sativa* L.) germplasm using microsatellite markers. Theoretical and Applied Genetics, 114, 559–568. 10.1007/s00122-006-0457-1 17136372

[tpg270336-bib-0033] Wang, M. , Zhu, Z. , Tan, L. , Liu, F. , Fu, Y. , Sun, C. , & Cai, H. (2013). Complexity of *indica‐japonica* varietal differentiation in Bangladesh rice landraces revealed by microsatellite markers. Breeding Science, 63, 227–232. 10.1270/jsbbs.63.227 PMC3688385 23853518

[tpg270336-bib-0034] Wang, W. , Mauleon, R. , Hu, Z. , Chebotarov, D. , Tai, S. , Wu, Z. , Li, M. , Zheng, T. , Fuentes, R. R. , Zhang, F. , Mansueto, L. , Copetti, D. , Sanciangco, M. , Palis, K. C. , Xu, J. , Sun, C. , Fu, B. , Zhang, H. , Gao, Y. , … Leung, H. (2018). Genomic variation in 3,010 diverse accessions of Asian cultivated rice. Nature, 557, 43–49. 10.1038/s41586-018-0063-9 PMC6784863 29695866

[tpg270336-bib-0035] Wang, Z. Y. , Zheng, F. Q. , Shen, G. Z. , Gao, J. P. , Snustad, D. P. , Li, M. G. , Zhang, J. L. , & Hong, M. M. (1995). The amylose content in rice endosperm is related to the post‐transcriptional regulation of the waxy gene. The Plant Journal, 7, 613–622. 10.1046/j.1365-313x.1995.7040613.x 7742858

[tpg270336-bib-0036] Weir, B. S. , & Cockerham, C. C. (1984). Estimating F‐statistics for the analysis of population structure. Evolution; Internation Journal of Organic Evolution, 38, 1358–1370. 10.2307/2408641 28563791

[tpg270336-bib-0037] Wunna , Watanabe, K. N. , Ohsawa, R. , Obara, M. , Yanagihara, S. , Aung, P. P. , & Fukuta, Y. (2016). Genetic variation of rice (*Oryza sativa* L.) germplasm in Myanmar based on genomic compositions of DNA markers. Breeding Science, 66, 762–767. 10.1270/jsbbs.16033 PMC5282748 28163592

[tpg270336-bib-0038] Xu, F. , Bao, J. , He, Q. , & Park, Y.‐J. (2016). Genome‐wide association study of eating and cooking qualities in different subpopulations of rice (*Oryza sativa* L.). BMC Genomics, 17, Article 663. 10.1186/s12864-016-3000-z PMC4992570 27544770

[tpg270336-bib-0039] Yang, L. , He, W. , Zhu, Y. , Lv, Y. , Li, Y. , Zhang, Q. , Liu, Y. , Zhang, Z. , Wang, T. , Wei, H. , Cao, X. , Cui, Y. , Zhang, B. , Chen, W. , He, H. , Wang, X. , Chen, D. , Liu, C. , Shi, C. , … Shang, L. (2025). GWAS meta‐analysis using a graph‐based pan‐genome enhanced gene mining efficiency for agronomic traits in rice. Nature Communications, 16, Article 3171. 10.1038/s41467-025-58081-1 PMC11968974 40180959

[tpg270336-bib-0040] Zaharia, M. , Bolosky, W. J. , Curtis, K. , Fox, A. , Patterson, D. , Shenker, S. , Stoica, I. , Karp, R. M. , & Sittler, T. (2011). Faster and more accurate sequence alignment with SNAP. arXiv. 10.48550/arXiv.1111.5572

[tpg270336-bib-0041] Zhao, K. , Tung, C. W. , Eizenga, G. C. , Wright, M. H. , Ali, M. L. , Price, A. H. , Norton, G. J. , Islam, M. R. , Reynolds, A. , Mezey, J. , McClung, A. M. , Bustamante, C. D. , & McCouch, S. R. (2011). Genome‐wide association mapping reveals a rich genetic architecture of complex traits in *Oryza sativa* . Nature Communications, 2, Article 467. 10.1038/ncomms1467 PMC3195253 21915109

[tpg270336-bib-0042] Zheng, X. , Levine, D. , Shen, J. , Gogarten, S. , Laurie, C. , & Weir, B. (2012). A high‐performance computing toolset for relatedness and principal component analysis of SNP data. Bioinformatics, 28, 3326–3328. 10.1093/bioinformatics/bts606 PMC3519454 23060615

