# Supplementary material for: Population structure and ecotype‐associated genomic differentiation in Bangladeshi rice germplasm (Oryza sativa L.)
Source: Plant Genome. 2026 Nov 11;19(4):e70336. doi: 10.1002/tpg2.70336 (PMC13755076; doi:10.1002/tpg2.70336)
Supplement: Supplementary file 1 [file TPG2-19-e70336-s001.pdf]

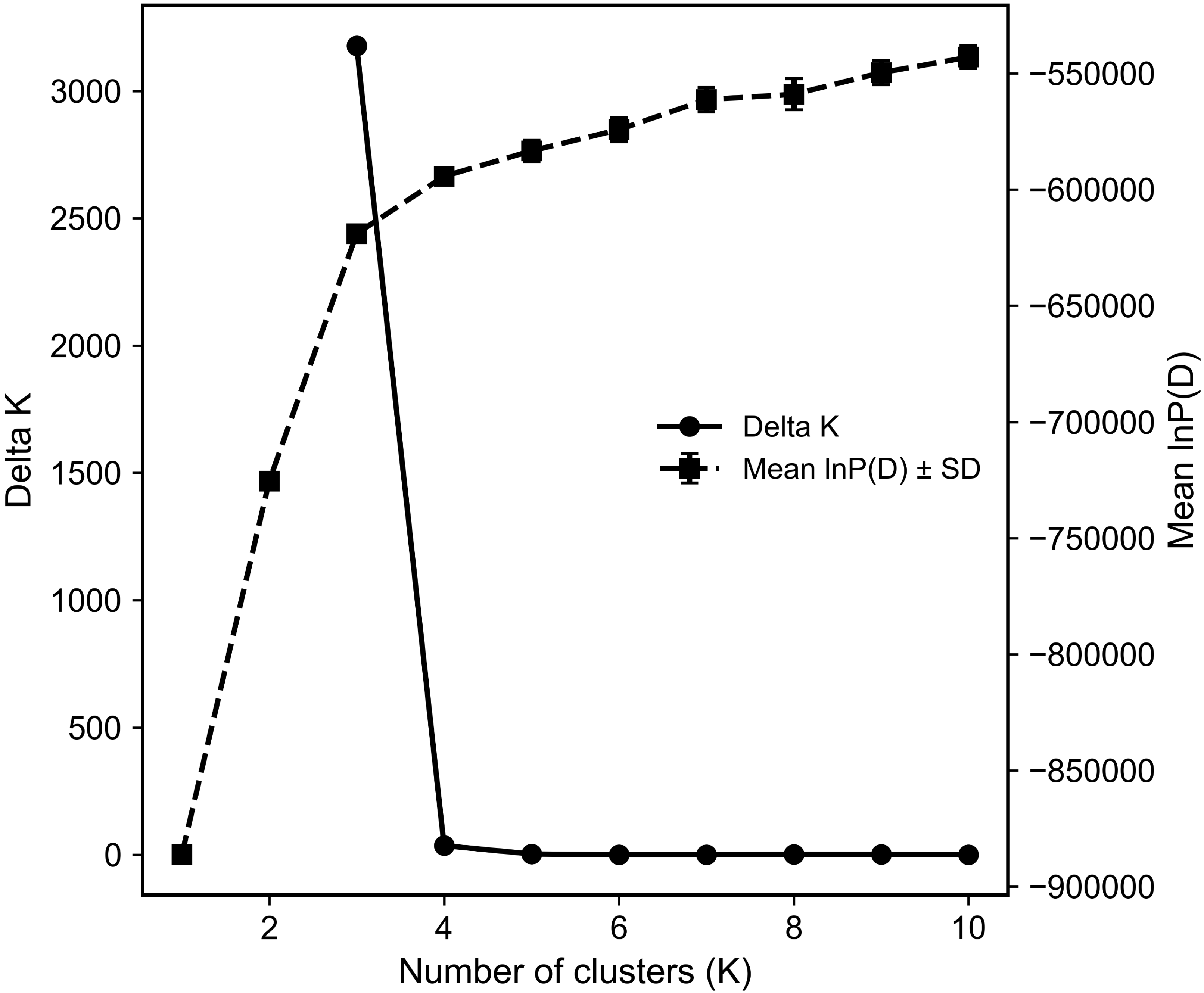

Delta K
Mean lnP(D) ± SD
Number of clusters (K)
Delta K
Mean lnP(D)
0
500
1000
1500
2000
2500
3000
2
4
6
8
10
−550000
−600000
−650000
−700000
−750000
−800000
−850000
−900000
