# Supplementary material for: Population structure and ecotype‐associated genomic differentiation in Bangladeshi rice germplasm (Oryza sativa L.)
Source: Plant Genome. 2026 Nov 11;19(4):e70336. doi: 10.1002/tpg2.70336 (PMC13755076; doi:10.1002/tpg2.70336)
Supplement: Supplementary file 2 [file TPG2-19-e70336-s002.pdf]

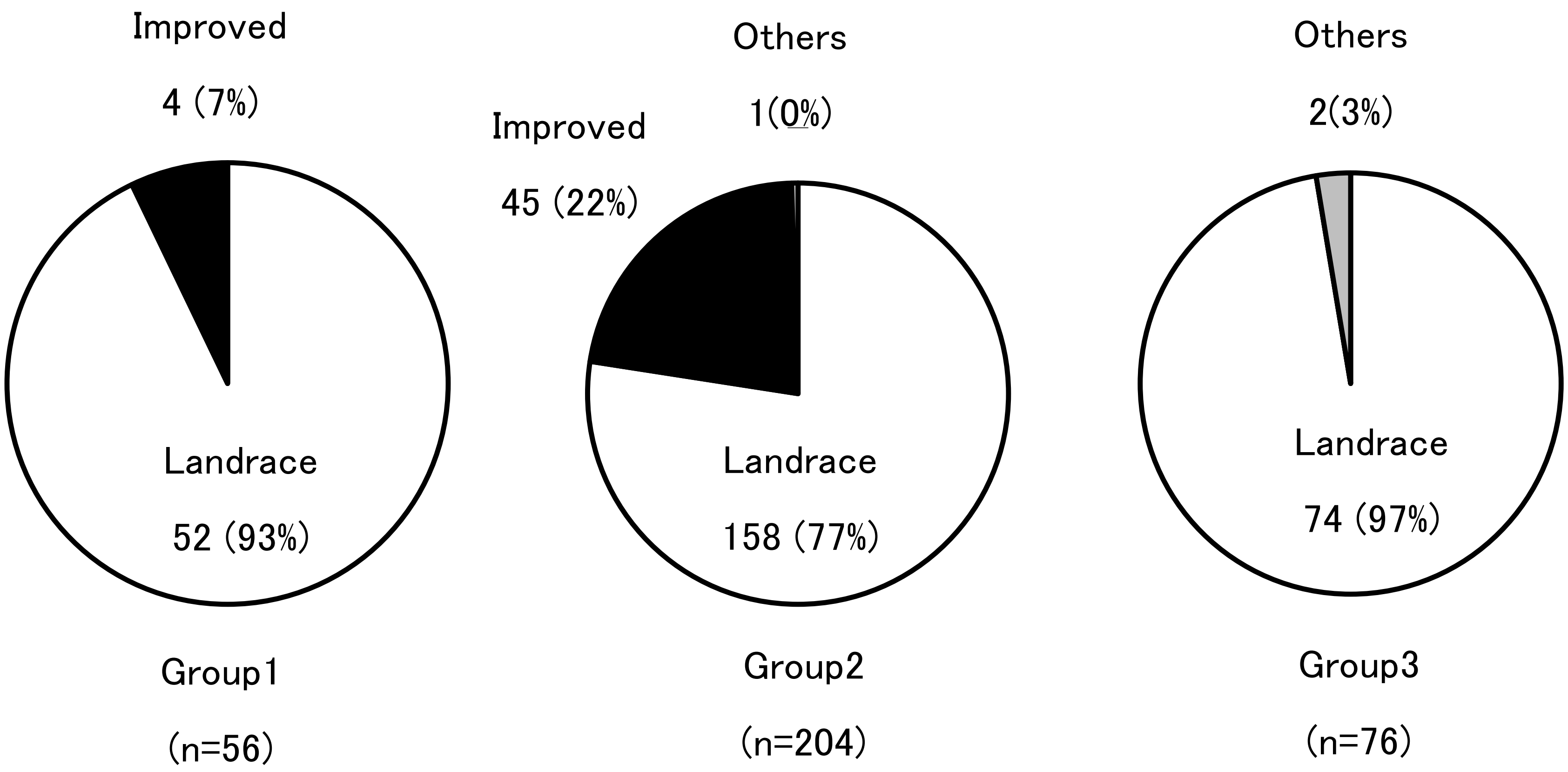
Improved
4 (7%)
Landrace
52 (93%)
Group1
(n=56)
Improved
45 (22%)
Others
1(0%)
Landrace
158 (77%)
Group2
(n=204)
Others
2(3%)
Landrace
74 (97%)
Group3
(n=76)
