# Supplementary material for: Population structure and ecotype‐associated genomic differentiation in Bangladeshi rice germplasm (Oryza sativa L.)
Source: Plant Genome. 2026 Nov 11;19(4):e70336. doi: 10.1002/tpg2.70336 (PMC13755076; doi:10.1002/tpg2.70336)
Supplement: Supplementary file 3 [file TPG2-19-e70336-s003.pdf]

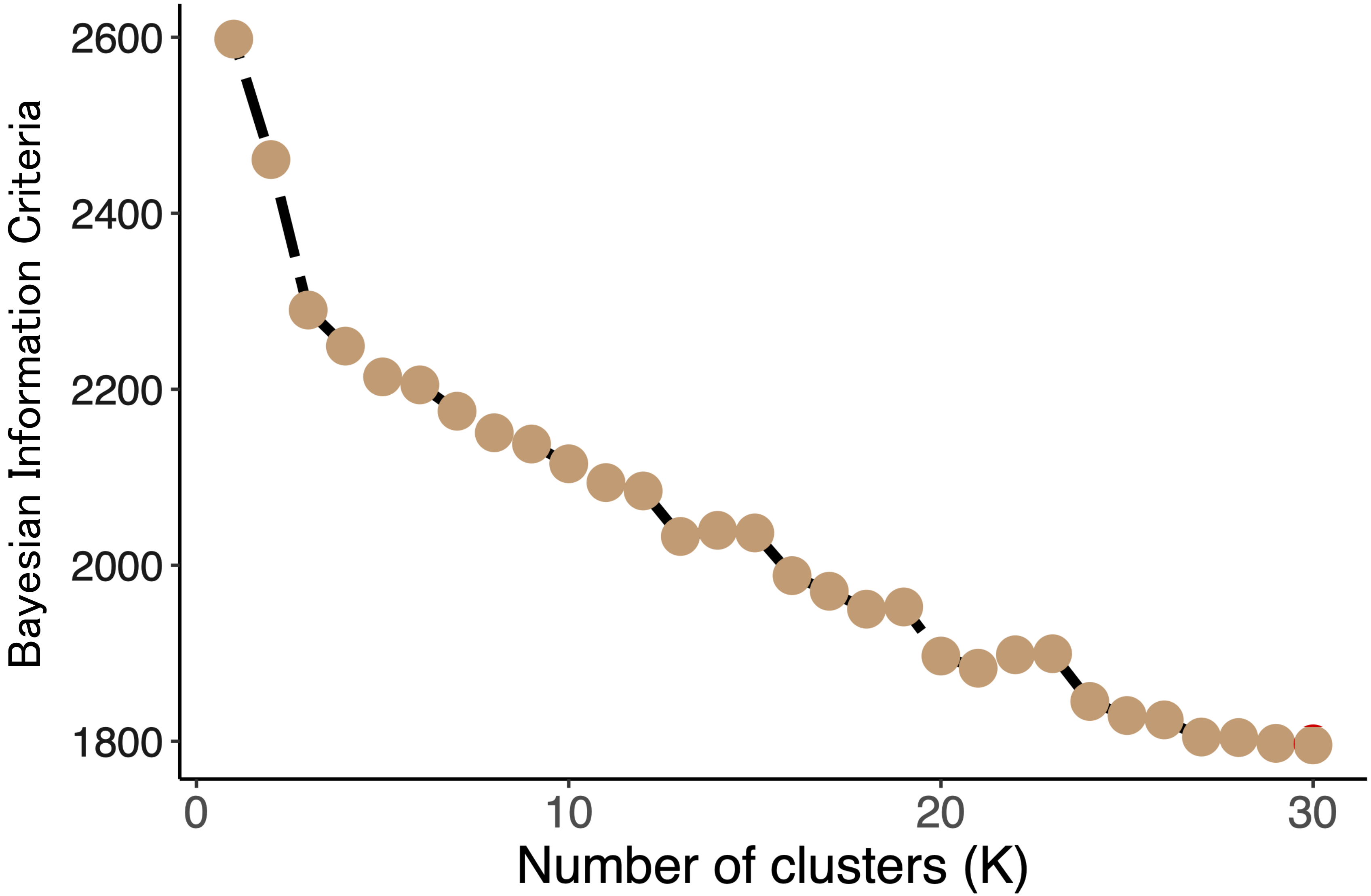

Bayesian Information Criteria
2600
2400
2200
2000
1800
0
10
20
30
Number of clusters (K)
